# Blood biomarkers in adults with lymph node enlargement contribute to diagnostic significance of malignancy

**DOI:** 10.18632/oncotarget.21963

**Published:** 2017-10-23

**Authors:** Shanshan Ma, Junbin Guo, Danlei Lu, Lixia Zhu, Meng Zhou, De Zhou, Li Li, Jingjing Zhu, Xiudi Yang, Yanlong Zheng, Xiujin Ye, Wanzhuo Xie

**Affiliations:** ^1^ Department of Hematology, the First Affiliated Hospital of Medical School of Zhejiang University, Zhejiang, Hangzhou, China; ^2^ Department of Hematology and Oncology, Wenling City First People's Hospital, Zhejiang, Wenling, China

**Keywords:** lymph node enlargement, lymphoma, cytokine, diagnosis, blood biomarker

## Abstract

Lymph node enlargement is a common presentation and has a possibility of malignancy like lymphoma that requires early diagnosis. This study aims to analyze the clinical characteristics of these patients and finds out useful predictors of malignant diseases. We retrospectively investigated 81 patients with lymph node enlargement between July 2, 2014 and May 17, 2016. The characteristics and laboratory findings were evaluated combining with the final diagnosis. The diagnoses were malignancy in 51 patients and benign lymphadenopathy in 30 patients. Increased beta2-microglobulin (B2M) (*P* = 0.012) was found to be associated with malignant diseases, and level of 3699.5 μg/L was used as a cut-off value to differentiate the malignancies from benign diseases, offering 63.4% sensitivity and 87.0% specificity. Immunoglobulin G (IgG) (*P* = 0.038) levels were significantly lower in malignant group, whose receiver operating characteristic curve showed that level of 1121.5 mg/dl had sensitivity and specificity as 58.5% and 82.6%. Moreover, through analysis of cytokines, we found interleukin-10 (IL-10) levels were elevated in malignant group compared with benign group. Serum B2M and IgG levels were concluded to be useful parameters for predicting malignancies. Besides, increased IL-10 levels indicated a higher risk of malignancy in some way.

## INTRODUCTION

Lymph node enlargement is a common problem in clinical settings, occurring in all ages. Findings from a Dutch study revealed an incidence of 0.6% in the general population [1]. Many diseases are associated with enlarged lymph nodes, such as infections, immune disorders, or malignancies [2]. In a previous study, the prevalence of malignancies was 11.5% among 11284 patients, and cancer risk was higher in males and the elderly [3]. Lymphoma, which usually presents with lymph node enlargement and fever, requires early and accurate diagnosis. Recently, lymphoma especially non-Hodgkin lymphoma (NHL) has been a significant cause of mortality worldwide, with an estimate 200000 deaths every year [4]. Even if precision therapy is available, long-term survival rates range from > 80% for Hodgkin's lymphoma (HL), 60% for diffuse large B-cell lymphoma (DLBCL) and to < 30% for peripheral T-cell lymphoma (PTCL) [5, 6, 7]. The critical task that we have to face is to differentiate benign lymphadenopathy from malignant diseases like lymphoma. Many factors including age of patients, associated signs and symptoms and the location of enlarged lymph nodes are involved with the final diagnosis. The advocated gold standard approach for the primary diagnosis of lymphoma is excisional lymph node biopsy [8]. Before that, a detailed history and necessary laboratory tests may help us in disease evaluation. Some variables including cytopenia and lactate dehydrogenase (LDH) levels were reported to be useful in predicting malignant diseases [9]. In addition, imaging modalities, such as ultrasonography (US) and positron emission tomography-computed tomography (PET-CT) are helpful to speculate malignant lesions. Blood biomarkers are more appealing due to the simplicity of obtaining blood samples. There are several serum biomarkers that are routinely used in clinical oncology, such as cancer antigen (CA)-125 for ovarian cancer [10]. However, lack of accurate blood biomarkers for initial diagnosis in patients with malignant lymph node enlargements still troubles physicians. We therefore developed this study to find some blood biomarkers to assess cancer risk among patients diagnosed with lymph node enlargement.

## RESULTS

### Patient characteristics

A total of 81 patients with lymph node enlargement were enrolled in our study. The clinical characteristics of all the patients were listed in Table 1 The primary features of these patients were prolonged lymph node enlargement (81/81), fever (66/81) and splenomegaly (47/81). Of these patients, 44 were males and 37 were females with a median age of 46 at initial diagnoses. Diagnoses included malignant diseases (51, including 47 with lymphoma and 4 with solid tumor) and benign diseases (30, including lymphadenitis, granuloma inflammation, reactive lymph node). The detailed pathologic diagnoses of malignant group were shown in Table 2.

**Table 1:** Baseline characteristics of all patients

	Total (*n* = 81)	Tumor (*n* = 51)	Non-tumor (*n* = 30)	*P* value
Variable	Median (range)			
Age at diagnosis (year)	46	51 (18–80)	36 (18–75)	0.003
Sex (M/F)	44/37	34/17	10/20	0.003
HGB (g/L)	102	97 (22–150)	116 (38–162)	0.018
PLT (1 × 10^9^/L)	113	103 (10–463)	138 (23-–710)	0.067
WBC (1 × 0^9^/L)	4.9	4.6 (0.5–19.6)	6.1 (0.8–31.6)	0.211
NEUT (1 × 10^9^/L)	2.7	2.9 (0–12.3)	2.2 (0.1–29.9)	0.570
TB (μmol/L)	11	16 (4–211)	9 (3–100)	0.001
TG (mmol/L)	1.64	1.99 (0.46-4.65)	1.28 (0.48–6)	0.001
Scr (μmol/L)	57	66 (29–116)	53 (30–97)	0.040
LDH (IU/L)	485	547 (144–13537)	479 (162–2762)	0.658
ALT (U/L)	26	32 (5–815)	26 (1–1444)	0.769
AST (U/L)	45	46 (11–482)	47 (10–1232)	0.621
CA-125 (U/ml)	30.2	51.3 (7.6–340.5)	25 (5.1–2208)	0.033
CRP (mg/L)	31.4	36.5 (0.4–198.2)	26.4 (0.1–338.1)	0.462
ESR (mm/h)	21	20 (2–104)	22 (2-99)	0.575
Fer (μg/L)	1096.5	1321.5 (18.5–42468.3)	410.8 (50.7–108802)	0.594
Fib (g/L)	2.54	2.54 (0.15–6.66)	2.56 (1.38–5.3)	0.168
Splenomegaly (Yes/No)	47/34	36/15	11//19	0.372
Fever (Yes/No)	66/15	42//9	24//6	0.792

*P* value: Comparisons between malignant and benign groups. HGB: hemoglobin, PLT: platelet, WBC: white blood cell, NEUT: neutrophilic granulocyte, ALT: alanine aminotransferase, AST: aspartate aminotransferase, Scr: serum creatinine, TG: triglyceride, LDH: lactate dehydrogenase, TB: total bilirubin, CA125: cancer antigen125, Fer: ferritin, Fib: fibrinogen, CRP:C reactive protein, ESR: erythrocyte sedimentation rate.

**Table 2:** The pathological diagnosis of malignant group

Pathological diagnosis	Number(n)
Diffuse large B cell lymphoma	13
Angioimmunoblastic T cell lymphoma	5
Extranodal natural killer/ T Cell Lymphoma	6
Follicular lymphoma	2
Hodgkin lymphoma	2
T Cell lymphoblastic lymphoma	2
Acute lymphoblastic leukemia(T)	2
Chronic lymphocytic leukemia	2
Mantle cell lymphoma	1
Anaplastic large-cell lymphomas	1
Non-Hodgkin lymphoma(T,unknown subtype)	9
Non-Hodgkin lymphoma(B,unknown subtype)	2
Solid tumor	4

### Serum Th1/Th2/Th17 cytokine levels in patients

**Figure 1:**
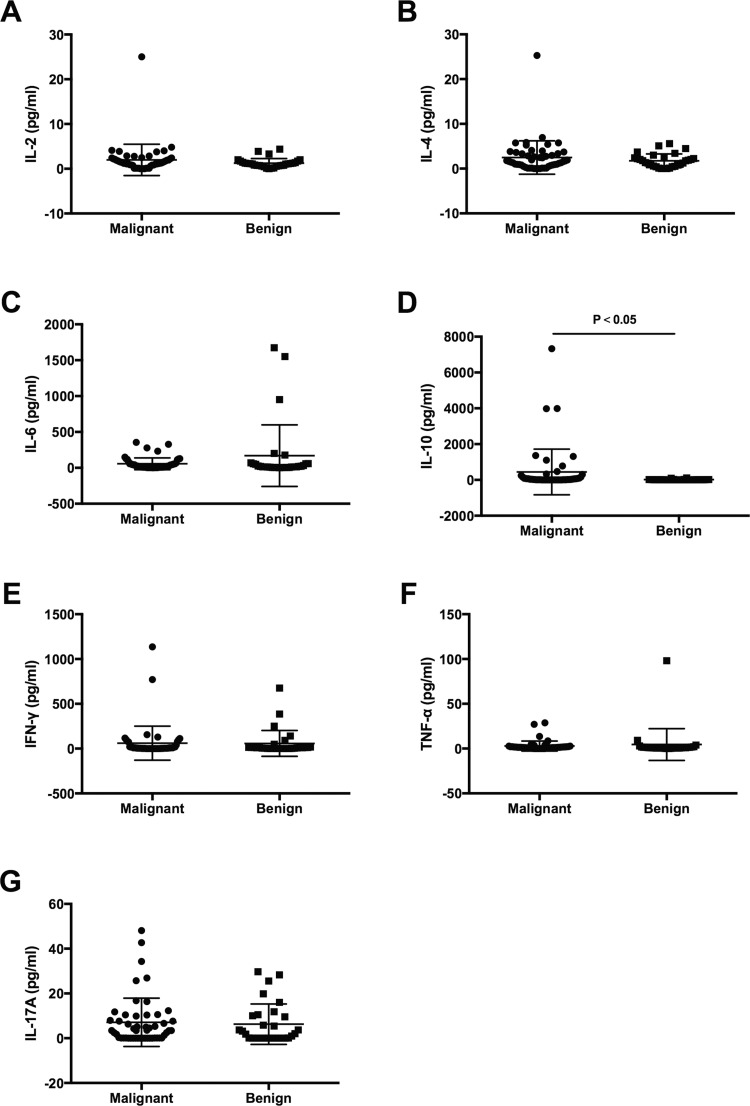
Comparisons of serum cytokine concentrations (pg/ml) among malignant and benign groups (**A**) IL-2; (**B**) IL-4; (**C**) IL-6; (**D**) IL-10; (**E**) IFN-y; (**F**) TNF-a; (**G**) IL-17A.

The serum Th1/TH2/Th17 cytokines (IL-2, IL-4, IL-6, IL-10, TNF-α, IFN-γ, IL-17A) were determined in all 81 patients. The IL-2, IL-4, IL-6, IL-10, TNF-α, IFN-γ and IL-17A levels for malignant group were 1.29 (0.01–25), 1.54 (0.01–25.28), 23.54 (1.82–356.02), 32.18 (1.44–7328), 1.41 (0.01–28.8), 5.78 (0.01–1135.96) and 3.46 (0.01–48.11) pg/ml, respectively (Table 3). However, in benign group, the IL-2, IL-4, IL-6, IL-10, TNF-α, IFN-γ and IL-17A levels were 1.09 (0.01–4.38), 1.55 (0.01–5.58), 16.85 (0.77–1673.33), 11.03 (0.49–113.92), 0.86 (0.01–98.01), 9.07 (0.01–675.85) and 1.92 (0.01–29.73), respectively. The cytokine pattern differed between the malignant and benign group (Table 3 and Figure 1). That is to say, the cytokine levels except IL-10 were comparable between the two groups (all *P* < 0.05). It is also worth mentioning that IL-10 level of the malignant group was significantly higher than the benign group (*P* < 0.05) =, even though it did not make sense in the multivariate analysis due to other clinical factors.

**Table 3:** Median levels, range and univariate analysis of cytokines by group

Groups	IL-2 (pg/ml)	IL-4 (pg/ml)	IL-6 (pg/ml)	IL-10 (pg/ml)	TNF-α (pg/ml)	IFN-γ (pg/ml)	IL-17A (pg/ml)
Malignant	1.29 (0.01–25)	1.54 (0.01–25.28)	23.54 (1.82–356.02)	32.18 (1.44–7328)	1.41 (0.01–28.8)	5.78 (0.01–1135.96)	5.78 (0.01–1135.96)
Benign	1.09 (0.01–4.38)	1.55 (0.01–5.58)	16.85 (0.77–1673.33)	11.03 (0.49–113.92)	0.86 (0.01–98.01)	9.07 (0.01–675.85)	1.92 (0.01–29.73)
*P* value	0.299	0.335	0.14	0.043	0.551	0.946	0.717

### Independent predicted factors for malignancy

In the multivariate analysis (Table 4), we can see that increased B2M (*P* = 0.012) and decreased IgG (*P* = 0.038) levels were independent predictors of malignancy. TB (*P* = 0.284), TG (*P* = 0.356), IL-10 (*P* = 0.065) and Scr (*P* = 0.870) levels were not independent factors to help diagnosing the malignancy.

**Table 4:** Multivariate analysis to identify independent predictors (*n* = 81)

	TB	TG	B2M	IgG	IL-10	Scr
*P* value	0.284	0.356	0.012	0.038	0.065	0.870
Odds ratio (95% Cl)	/	/	1.001 (1.000–1.001)	0.999 (0.998–1.000)	/	/

*P* value: Comparisons between malignant and benign groups.

**Figure 2:**
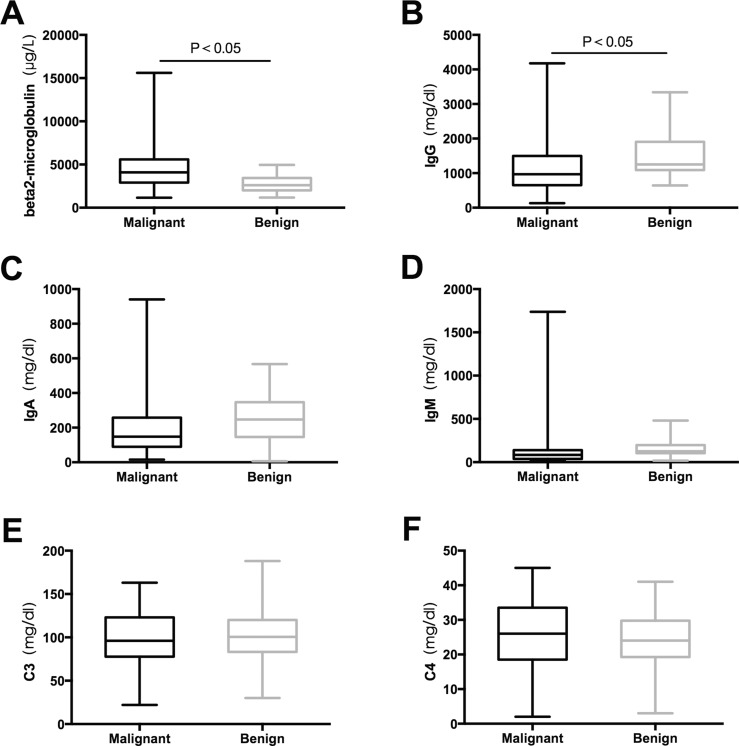
Comparisons of serum beta2-microglobulin and immunoglobulin concentrations among malignant and benign groups (**A**) beta2-microglobulin, (**B**) lgG, (**C**) lgA, (**D**) lgM, (**E**) C3, (**F**) C4.

We totally detected IgG, IgA, IgM, C3 and C4 in all the patients. The great difference between the malignant and benign group was seen in terms of IgG, IgA and IgM levels that were consistently low in malignant diseases (Figure 2), whereas the decrease of IgG levels were significant. In contrast, C3 and C4 levels showed a vastly different tendency.

**Figure 3:**
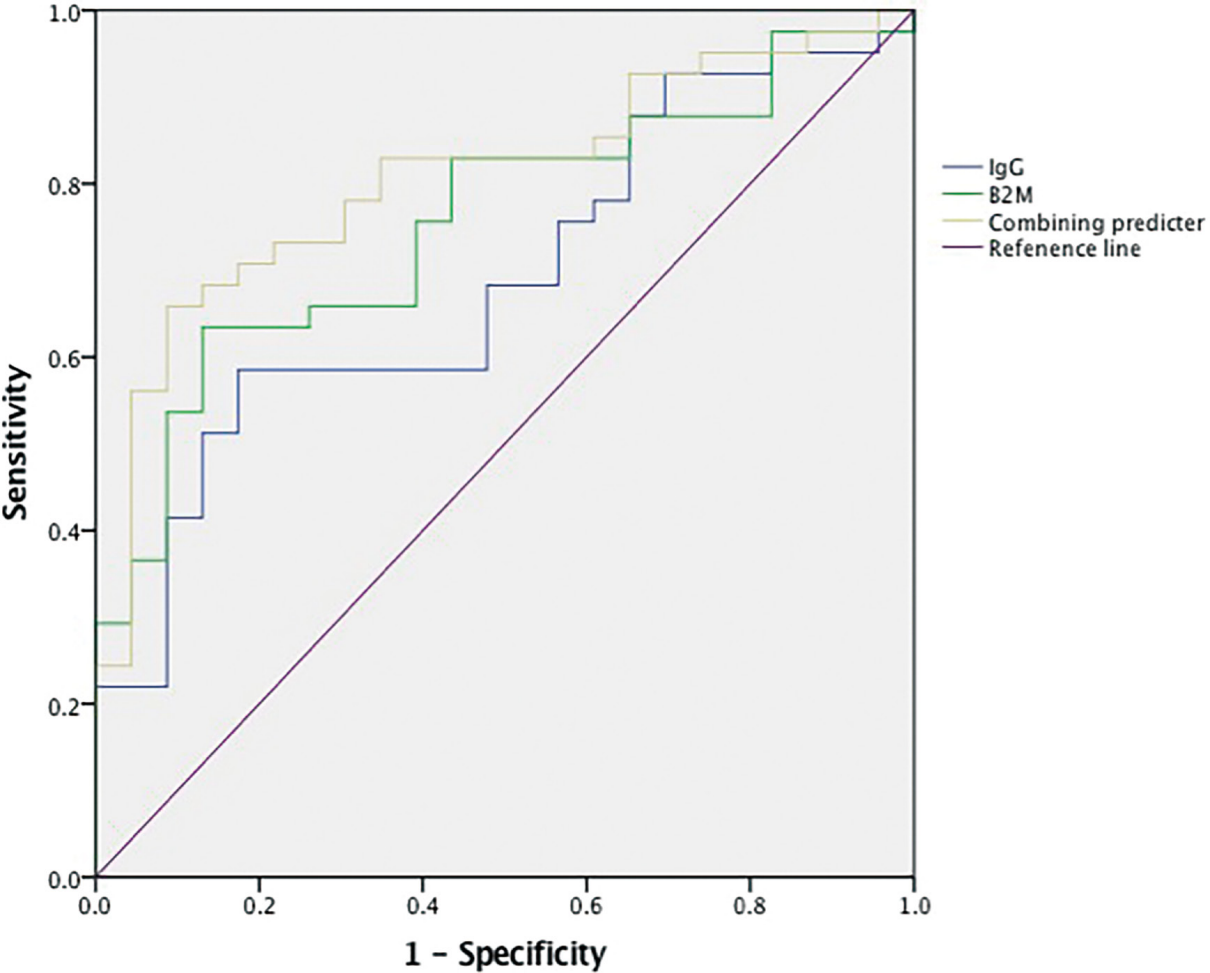
Roc curves of B2M, lgG and combining predicter between maglinant and benin groups The diagonal line is the reference line. B2M:beta2-microglobulin. leG:immunoglobulin G.

The area under the ROC curve is an appropriate measure for describing the overall accuracy of a diagnostic test, and higher area under curve (AUC) value means better diagnostic value. ROC curves were generated based on the B2M, IgG levels and combining predictors for malignant and benign patients (Figure 3). AUC values for B2M and IgG were 0.756 (95% Cl, 0.558–0.820) and 0.689 (95% Cl, 0.6380.874) respectively. Based on the Youden index, ROC curve of B2M showed that 3699.5 μg/L had sensitivity and specificity of 63.4% and 87.0%. Furthermore, ROC curve of IgG showed that 1121.5mg/dl had sensitivity and specificity of 58.5% and 82.6%. We got the combining predictor through Logistic regression equation (Y = B2M-IgG). AUC value for combining predictor was 0.808 (95% Cl, 0.701–0.915), indicating that it had larger diagnostic value. With the same method, we defined the cut-off point to be 2541, with sensitivity and specificity of 65.9% and 91.3%. Taken the results together, we proposed that the patients who had continued lymph node enlargement with B2M above 3699.5 μg/L, IgG below 1121.5 mg/dl and Y above 2541 had a higher chance to be malignant.

## DISCUSSION

In patients with lymph node enlargement, a fast, convenient, and accurate diagnosis is needed promptly. Despite many patients appear to be benign; the most worrisome situation we may face is that there is always a possibility of a malignancy. To the best of our knowledge, few reports have examined methods for determining blood biomarkers for the prediction of malignant diseases before biopsy. Through this retrospective study, we have concluded that in patients presenting with lymph node enlargement, levels of cytokines, B2M and IgG can be used to predict malignancies.

Clinically, many benign lymphadenopathies are self-limited and require no treatment [11]. In our study, most patients had fever and splenomegaly at the same time, which may prompt them to our department. In a way, these patients were likely to have malignancies like lymphomas. Consequently, 63% (51/81) patients were found to have tumors while 37% (30/81) were benign diseases. Interestingly, in the malignant group, almost all had hematologic malignancies except for 4 patients. We focused on patients who came to hospital mainly because of lymph node enlargement with or without fever, which frequently occurs in hematologic malignancy. However, in solid tumor like lung cancer, patient usually has more obvious clinical manifestations such as cough and chest pain. Besides, lymph node metastasis always happens in advanced stage of the disease. All of the above may account for the disease distribution of malignancies in our study.

IL-10, which comes from B-lymphocytes, is an anti-inflammatory cytokine that inhibits macrophages and downgrades cytokine production by T cells [12]. Usually, it serves as a marker of a state of inflammatory activity. We detected 7 cytokines of all 91 patients and determined that increased IL-10 levels were associated with higher risk of malignancy, especially lymphoma by univariate analysis. There is no doubt that it remains as the marker of inflammatory process in benign lymphadenopathies such as reactive lymph node or lymphadenitis, in which the mean IL-10 level was also increased. However, IL-10 levels were significantly higher in the malignant group. This finding was consistent with Edlefsen’study about 491 B-cell NHL cases [13]. The nested case-control study found an increased risk of B cell NHL in women with increased levels of IL-10. Nevertheless, it is to be observed that inheritance of the IL10-3575 T to A polymorphism has been associated with increased risk of NHL, with a particularly strong association with DLBCL, which resulted in reduction of IL-10 [14]. Some evidences also exist that showed the NHL cells themselves as well as other normal nonmalignant infiltrating cells contributed to the increased IL-10. Besides, IL-2, IL-6, IL-10 and TNF-αmay cooperate to increase neoplastic lymphocytes proliferation [15]. Overall, polymorphism of gene, serum IL-10 levels and risk of lymphoma make up a complex network. For the limitations of experimental conditions, we did not measure other cytokines. In patients with Hodgkin lymphoma, IL-2R levels were significantly higher than the non-lymphoma controls and were treated as an independent prognostic factor [16]. Interestingly enough, IL-1RA and IL-2Rαlevels showed similar performance in T cell lymphoma [17]. Thus, it can be seen that further study would be required to better understand the relationship between cytokines and the pathogenesis of lymphoma.

The results of our study demonstrated that serum B2M levels were markedly elevated in malignancy in multivariate models including other factors such as TB, TG, IgG, IL-10 and Scr. This suggested that assessment of serum B2M might provide evidence of malignant diseases. Furthermore, 3699.5 μg/L as cut-off point had better performance in prediction of malignancy in ROC curve analysis. As we know, B2M is a subunit of the human leukocyte antigen-class I (HLA-I), which is linked to immunologically and hematologically relevant molecules [18]. Serum B2M can be detected as a result of release from the cell surface or cytoplasm [18]. The ability of carcinoma cells to produce a higher concentration of beta2-microglobin than the non-neoplastic cells may be due to either active synthesis, increased cell breakdown, or both [19, 20]. Correlation between tumor burden and serum B2M has been previously suggested because serum B2M is frequently elevated in patients with lymphoma [21]. This was also supported by our study. Albitar examined the clinical relevance of HLA-I and B2M levels in NHL (*n* = 65) and HL (*n* = 37). Finally, NHL and HL patients had significantly higher levels of sHLA-1 and B2M than control subjects [22]. In another study, increased serum B2M levels has been suggested as a powerful prognostic factor in extranodal natural killer/T cell lymphoma [23]. All of the above suggests that B2M may reflect a biological process that represents tumor mass and immune response to the malignancy.

It is generally known that immunoglobulin production depends on the normal B and T-cell interactions *in vivo*. NHL is a tumor of the immune system causing the alteration of immune function, but its specific relationship is complex. By inference, immunoglobulin levels may change in patients with NHL. We observed that hypogammaglobulinaemia was the distinct aberration in the malignant group, followed by significant decrease of IgG levels (*P* < 0.05 =. Level of 1121.5 mg/dl had sensitivity and specificity of 58.5% and 82.6%, which had the best reliability of diagnosis. In a population-based case-control study, IgG levels were also reduced in NHL cases [24]. Moreover, low serum IgG levels were biomarkers of prognosis that were associated with short survival [25]. Similar result could also be seen in the study of chronic lymphocytic leukemia (CLL). CLL is a kind of disease that has varied clinical features, often complicated by hypogammaglobulinaemia [26]. However, the prevalence of hypogammaglobulinemia in CLL varies considerably in the reported literature, ranging from 27% [26] to 55% [27]. In these patients, the levels of normal B lymphocyte are usually downgraded, contributing to the blockage of antibodies production. Therefore, immunoglobulin replacement therapy has well documented benefit for patients with CLL with hypogammaglobulinemia to reduce recurrent bacterial infections [26]. Furthermore, this therapy may also be feasible in other lymphomas when the patient has a low level of IgG. Besides hematological malignancies, solid tumor like pancreatic cancer also manifested with a changed immunoglobulin level. In a recent study, investigators found that the level of IgG expression varied depending on the stages of the pancreatic cancer with more malignant cancers expressing more IgG [28]. The difference between the two kinds of tumors probably resulted from different etiologies and pathogenesis. It was a pity that we had not collected the data of IgE level as Melbye reported that people with detectable serum IgE had a reduced risk of NHL [29]. All in all, we believe that immunoglobulin levels, especially decreased IgG level, has positive prediction of malignant lymphadenopathy.

We have got a regression equation (Y = B2M-IgG) through multivariate analysis and regarded it as a combining predictor. On the bright side, area under the ROC curve for combining predictor was 0.808, which was superior to others, indicating that it had larger diagnostic value. When the Youden index came max, we defined the cut-off value of 2541, showing a sensitivity of 65.9% and a specificity of 91.3%. This combined biomarkers demonstrated improved accuracy than these tested individually. We hope the new index can help more in the differential diagnosis of lymphadenopathy.

In conclusion, the lymph node biopsy remains the advocated gold standard approach for the diagnosis of lymph node enlargement. Our study determined several important parameters to assess risk of malignancy among patients with lymph node enlargement. Decreased IgG and increased B2M showed higher risk of malignancy. In addition, cytokine like IL-10 plays an important role in the development of malignancy. To some extent, the risk estimates for malignancy may offer clinicians a tendency of diagnosis and guidance on the necessity of biopsy.

## MATERIALS AND METHODS

### Ethical approval

All patients provided informed consent in accordance with requirements of the Declaration of Helsinki. Besides, all experimental protocols were approved by the Ethics Committee of the First Affiliated Hospital of Medical School of Zhejiang University and were performed in accordance with the approved guidelines of Zhejiang University.

### Patients

Between July 2, 2014 and May 17, 2016, 91 adult patients with lymph node enlargements admitted to the First Affiliated Hospital of Medical School of Zhejiang University were enrolled in the study. Eventually, 10 patients were excluded because they did not have pathological biopsy results. All the patients presented with lymph node enlargements, including superficial and intra-abdominal, and most of which had fever of unknown origin. Fever was defined as maximum temperature reading of > 38.5°C. Patients were examined, blood samples were taken for various analyses, and serum cytokine levels were obtained when they were admitted. No patients in this cohort had received long time corticosteroid treatment or chemotherapy before serum samples were taken for measurement.

### Laboratory methods

Peripheral blood samples of all the patients were collected at the time of admission. We performed various test including blood routine test [hemoglobin (HGB), platelet (PLT), white blood cell (WBC) and neutrophilic granulocyte (NEUT)], biochemical test [albumin (ALB), alanine aminotransferase (ALT), aspartate aminotransferase (AST), serum creatinine (Scr), triacylglyceride (TG), lactate dehydrogenase (LDH), and total bilirubin (TB)], immunoglobulin (IgG, IgM, IgA), complement (C3, C4), cancer antigen125 (CA125), ferritin (Fer), fibrinogen (Fib), erythrocyte sedimentation rate (ESR), beta2-microglobulin (B2M), cytokine [interleukin-2 (IL-2), interleukin-4 (IL-4), interleukin-6 (IL-6), interleukin-10 (IL-10), interleukin-17A (IL-17A)], tumor necrosis factor-α(TNF-α) and interferon-γ(IFN-γ). Concentrations of cytokines were quantitatively determined with use of the cytometric bead array(CBA) kit (CBA Human Th1/Th2/Th17 Kit ; BD Biosciences, San Jose, California) as described in the literature [30]. And the minimal and maximum limits of detection for all 7 cytokines were 1 pg/mL and 5000 pg/mL, respectively.

### Statistical analysis

Serum concentrations of individual laboratory tests were compared between groups using Pearson's chi-square test or Mann-Whitney *U* test for bivariate correlation analysis. We used the presence or absence of malignancy as dependent variables, while univariate Logistic regression analysis was used to study the tumor-related factors. The variables that were assessed to be significant in univariate regression analysis were entered as independent variables in multivariate logistic regression analysis, thereby forming the regression model. Receiver operating characteristic (ROC) curve was derived from the B2M, IgG levels and combining predictors of the malignant and benign patients. All statistical analyses were performed using SPSS 23.0 software (SPSS Inc, Chicago, IL). A value of *P* < 0.05 was considered to be statistically significant.
